# Supplementary material for: Pharmacological treatment of depression: A systematic review comparing clinical practice guideline recommendations
Source: PLoS One. 2020 Apr 21;15(4):e0231700. doi: 10.1371/journal.pone.0231700 (PMC7173786; doi:10.1371/journal.pone.0231700)
Supplement: S1 Appendix — (DOCX) [file pone.0231700.s001.docx]

**S1 Appendix. Systematic review strategy: research terms used to identify clinical practice guidelines in the Medline, Embase, and the Cochrane Library databases.**

**Medline** (PubMed website)—publication date from 2011/01/01 to 2016/12/31

| (((((("Guideline" [Publication Type] OR "Guidelines as Topic"[Mesh] OR "Practice Guideline" [Publication Type] OR "Health Planning Guidelines"[Mesh]) OR "Clinical Protocols"[Mesh])) OR ("Consensus Development Conference, NIH" [Publication Type] OR "Consensus Development Conference" [Publication Type] OR "Consensus"[Mesh]))) OR "Standard of Care"[Mesh])) "Guideline" [Publication Type] OR "Guidelines as Topic"[Mesh] OR "Practice Guideline" [Publication Type] OR "Health Planning Guidelines"[Mesh]) OR "Clinical Protocols"[Mesh])) OR ("Consensus Development Conference, NIH" [Publication Type] OR "Consensus Development Conference" [Publication Type] OR "Consensus"[Mesh]))) OR "Standard of Care"[Mesh])))))) AND (("Depressive Disorder"[Mesh] OR "Depressive Disorder, Major"[Mesh] OR Depressive Disorders OR Disorder, Depressive OR Disorders, Depressive OR Neurosis, Depressive OR Depressive Neuroses OR Depressive Neurosis OR Neuroses, Depressive OR Depression, Endogenous OR Depressions, Endogenous OR Endogenous Depression OR Endogenous Depressions OR Depressive Syndrome OR Depressive Syndromes OR Syndrome, Depressive OR Syndromes, Depressive OR Depression, Neurotic OR Depressions, Neurotic OR Neurotic Depression OR Neurotic Depressions OR Melancholia OR Melancholias OR Unipolar Depression OR Depression, Unipolar OR Depressions, Unipolar OR Unipolar Depressions)) |
| --- |

**EMBASE**—publication date from 2011/01/01 to 2016/12/31

| **#1 'practice guideline'**/mj OR **'consensus development'**/exp/mj OR **'clinical protocol'**/mj  **#2 'depression'**/exp  #3 #1 AND #2 |
| --- |

**Cochrane Library**

| ((**practice guidel’ne**/mj OR ‘**consensus development**’/exp/mj OR ‘**clin’cal protocol’**/mj AND (‘**depression**’/exp) AND (**2011**:py OR **2012**:py OR **2013**:py OR **2014**:py OR **2015**:py OR **2016**:py AND [embase]/lim)  #6 #4 AND #5 |
| --- |
