# Supplementary material for: Pharmacological treatment of depression: A systematic review comparing clinical practice guideline recommendations
Source: PLoS One. 2020 Apr 21;15(4):e0231700. doi: 10.1371/journal.pone.0231700 (PMC7173786; doi:10.1371/journal.pone.0231700)
Supplement: S2 Appendix — (DOCX) [file pone.0231700.s002.docx]

**S2 Appendix.** **Reasons for the inclusion and exclusion of clinical practice guidelines**.

| **References of the excluded Guideline** | **Reasons for exclusion** |
| --- | --- |
| Institute for Clinical Systems Improvement. Adult depression in primary care. [Bloomington, MN]: ICSI; 2016. | Duplicate |
| Austin M-P, Highet N and the Expert Working Group. Mental Health Care in the Perinatal Period: Australian Clinical Practice Guideline. Melbourne: Centre of Perinatal Excellence; 2017. | Duplicate |
| Grinspun, D, Bajnok I, Rey M.Delirium, Dementia, and Depression in Older Adults: Assessment and Care. Toronto, ON: Registered Nurses' Association of Ontario; 2016. | Duplicate |
| National Guideline Clearinghouse. Delirium, dementia, and depression in older adults: assessment and care. In: [Internet]. Rockville, MD: Agency for Healthcare Research and Quality (AHRQ); 2016 Jul 01 [cited 2017 Jan 19]. Available from: https://www.guideline.gov. | Duplicate |
| National Institute for Health and Care Excellence. Depression in adults: recognition and management. 2009 October [cited 2017 June 30; Internet]. Available from: https://www.nice.org.uk/guidance/cg90/evidence. | Duplicate |
| Boltz M, editor. Evidence-based geriatric nursing protocols for best practice. New York: Springer; 2012. | Duplicate |
| Depression. University of Michigan Health System. NGC:008672. | Duplicate |
| Álvarez Ariza M, **Atienza Merino** G, Ávila González MJ, González García A, Guitián Rodrígue D. GPC sobre el Manejo de la Depresión en el Adulto. [n.p.]: Ministerio de Sanidad, Servicios Sociales e Igualdad; 2014. | Duplicate |
| National Guideline Clearinghouse. Depression (Singapore) [Internet]. Rockville, MD: Agency for Healthcare Research and Quality (AHRQ); 2012 Jan 1 [cited 2016 Oct 19]. Available from https://www.guideline.gov/summaries/summary/39324. | Duplicate |
| Austin M-P, Highet N and the Expert Working Group. Mental Health Care in the Perinatal Period: Australian Clinical Practice Guideline. Melbourne: Centre of Perinatal Excellence; 2017. | Duplicate |
| McDermott B, Baigent M, Chanen A, Fraser L, Graetz B, Hayman N, Newman L, Parikh N, Peirce B, Proimos J, Smalley T, Spence S; beyondblue Expert Working Committee. Clinical practice guidelines: Depression in adolescents and young adults. Melbourne: Agency for Healthcare Research and Quality; 2010. | Duplicate |
| Michigan Quality Improvement Consortium Guideline. Primary care diagnosis and management of adults with depression [Internet]. [n.p.]: MQIC; 2018 [cited 2016 Oct 19]. Available from: http://mqic.org/guidelines.htm | Duplicate |
| National Institute for Health and Clinical Excellence. Depression in children and young people: identification and management in primary, community and secondary care. Leicester (UK): British Psychological Society; 2005. | Duplicate |
| Michigan Quality Improvement Consortium Guideline. Primary care diagnosis and management of adults with depression [Internet]. [n.p.]: MQIC; 2018 [cited 2016 Oct 19]. Available from: http://mqic.org/guidelines.htm | Duplicate |
| Connolly KR, Thase ME. If at first you don’t succeed: a review of the evidence for antidepressant augmentation, combination and switching strategies. Drugs. 2011 Jan 1;71(1):43-64. https://doi.org/10.2165/11587620-000000000-00000. | Duplicate |
| National Institute for Health and Care Excellence. Common mental health disorders. Identification and pathways to care. [London]: NICE; 2011. | Without pharmacological treatment |
| Grinspun, D, Bajnok I, Rey M.Delirium, Dementia, and Depression in Older Adults: Assessment and Care. Toronto, ON: Registered Nurses' Association of Ontario; 2016. | Without pharmacological treatment |
| Canadian Task Force on Preventive Health Care, Joffres M, Jaramillo A, Dickinson J, Lewin G, et al. Recommendations on screening for depression in adults. CMAJ. 185(9):775–82. https://doi.org/10.1503/cmaj.130403. | Without pharmacological treatment |
| Boltz M, editor. Evidence-based geriatric nursing protocols for best practice. New York: Springer; 2012. | Without pharmacological treatment |
| Patten SB. Updated CANMAT guidelines for treatment of major depressive disorder. Can J Psychiatry. 61(9):504-5. https://doi.org/10.1177/0706743716660034. | Without pharmacological treatment |
| Siu AL, US Preventive Services Task Force (USPSTF), Bibbins-Domingo K, Grossman DC, Baumann LC Davidson KW. Screening for Depression in Adults: US Preventive Services Task Force Recommendation Statement. JAMA. 315(4):380-7. https://doi.org/10.1001/jama, 2015.18392. | Language used |
| Thase ME. Recommendations for screening for depression in adults. JAMA. 315(4):349-50. https://doi.org/10.1001/jama.2015.18406. | Language used |
| Frye MA. Clinical practice: Bipolar disorder--a focus on depression. N Engl J Med. 2011 Jan 6;364(1):51-9. | Subject matter |
| Malhi GS, Bassett D, Boyce P. Bryant R, Fitzgerald PB, Fritz K, et al. Royal Australian and New Zealand College of Psychiatrists clinical practice guidelines for mood disorders. Aust N Z J. Psychiatry. 2015 Dec;49(12):1087-206. https://doi.org/10.1177/0004867415617657. | Subject matter |
| Andersen BL, DeRubeis RJ, Berman, BS, Gruman J, Champion VL, Massie MJ, et al. Screening, assessment, and care of anxiety and depressive symptoms in adults with cancer: an American Society of Clinical Oncology guideline adaptation. J Clin Oncol. 2014 May 20;32(15):1605-19. https://doi.org/10.1200/JCO.2013.52.4611. | Subject matter |
| American Medical Directors Association. Guideline summary: Depression in the long term care setting. Columbia, MD: AMDA; 2011. | Subject matter |
| Canadian Task Force on Preventive Health Care, Joffres M, Jaramillo A, Dickinson J, Lewin G, Pottie K, et al. Recommendations on screening for depression in adults. CMAJ. 2013 Jun 11;185(9):775-82. https://doi.org/10.1503/cmaj.130403. | Subject matter |
| Li M, Kennedy EB, Byrne N, Gérin-Lajoie C, Katz MR, Keshavarz H, et al. The management of depression in patients with cancer. J Oncol Pract. 2016 Aug;12(8):747-56. https://doi.org/10.1200/JOP.2016.011072 | Subject matter |
| Ostacher MJ, Tandon R, Suppes T. Florida best practice psychotherapeutic medication guidelines for adults with bipolar disorder: A novel, practical, patient-centered guide for clinicians. J Clin Psychiatry, 2016;77(7):920-6. https://doi.org/10.4088/JCP.15cs09841. Available in: http://www.embase.com/search/results?subaction=viewrecord&from=export&id=L611538719. | Subject matter |
| Pfennig A, Bschor T, Falkai P, Bauer M. The diagnosis and treatment of bipolar disorder: recommendations from the current s3 guideline. Dtsch Arztebl Int. 110(6):92-100. https://doi.org/10.3238/arztebl.2013.0092. | Subject matter |
| Rosenblat JD, McIntyre RS. Treatment recommendations for DSM-5–defined mixed features. CNS Spect. 2017 Apr;22(2):147-154. https://doi.org/10.1017/S1092852916000432. | Subject matter |
| Alexopoulos GS. Pharmacotherapy for late-life depression. J Clin Psychiatry. 2011 Jan;72(1):e04. https://doi.org/10.4088/JCP.7085tx2cj. | Not a CPG |
| Andreescu C, Reynolds CF 3rd. Late-life depression: evidence-based treatment and promising new directions for research and clinical practice. Psychiatr Clin North Am. 2011 Jun;34(2): 335-55, vii-iii. https://doi.org/10.1016/j.psc.2011.02.005. | Not a CPG |
| Arnow BA, Steidtmann D, Blasey C, Manber R, Constantino MJ, Klein DN, et al. The relationship between the therapeutic alliance and treatment outcome in two distinct psychotherapies for chronic depression. J Consult Clin Psychol. 2013 Aug; 81(4):627-38. https://doi.org/10.1037/a0031530. | Not a CPG |
| Ayub-Dargél A, Masson M, Henry C. The RANZCP guidelines: Managing mood disorders in the real world. Aust N Z J Psychiatry. 2016 Dec;50(12):1198-9. https://doi.org/10.1177/0004867416676373. | Not a CPG |
| Bland P. Raising standards of care for patients with depression. Practitioner. 2011 May;255(1740):21-5:3. | Not a CPG |
| Boffin N, Bossuyt N, Declercq T, Vanthomme K, Van Casteren V. Incidence, patient characteristics and treatment initiated for GP-diagnosed depression in general practice: Results of a 1-year nationwide surveillance study. Fam Prac.2012 Dec;29(6):678-87. https://doi.org/10.1093/fampra/cms024. | Not a CPG |
| Bohra HM, Novak M. Depression in patients with chronic kidney disease. CANNT J. 2015 Jul-Sep;25(3),34-8. | Not a CPG |
| Busch FN, Sandberg LS. Combined treatment of depression. Psychiatr Clin North Am. 2012 Mar;35(1):165-79. https://doi.org/10.1016/j.psc.2011.10.002. | Not a CPG |
| Cohen A. The QOF, NICE, and depression. Br J Gen Pract. 2011 Sep;61(590):549. https://doi.org/10.3399/bjgp11X593785. | Not a CPG |
| Baumann S, Benson-Martin J, Cossie Q, Gilfillan K, Horn N, Kaliski S. SASOP treatment guidelines for psychiatric disorders: Eminence or evidence based? S Afr J Psychiatr. 2019 Dec:20(2):a529. https://doi.org/10.4102/sajpsychiatry.v20i2.529. | Not a CPG |
| Cosgrove L, Shaughnessy AF, Wheeler EE, Austad KE, Kirsch I, Bursztajn HJ. The American Psychiatric Association’s guideline for major depressive disorder: A commentary. Psychother Psychosom. 2012;81(3):186-8. https://doi.org/10.1159/000335523. | Not a CPG |
| Cuijpers P. Effective therapies or effective mechanisms in treatment guidelines for depression? Depress Anxiety. 2013 Nov;30(11):1055-7. https://doi.org/10.1002/da.22205. | Not a CPG |
| Cuijpers P. Combined pharmacotherapy and psychotherapy in the treatment of mild to moderate major depression? JAMA Psychiatry. 2014 Jul 1:71(7):747-8. https://doi.org/10.1001/jamapsychiatry.2014.277. | Not a CPG |
| De Coteau PA, Byrne CD, Russell, V. The HSE/ICGP guidelines on the management of depression and anxiety disorders in primary care. Ir Med J. 2012 Jul-Aug;105(7):251. | Not a CPG |
| Desseilles M, Witte J, Chang TE, Iovieno N, Dording CM, el al. Assessing the adequacy of past antidepressant trials: A clinician’s guide to the antidepressant treatment response questionnaire. J Clin Psychiatry. 2011 Aug;72(8):1152-4. https://doi.org/10.4088/JCP.11ac07225. | Not a CPG |
| Canadian Agency for Drugs and Technologies in Health. Diagnosing, Screening, and Monitoring Depression in the Elderly: A Review of Guidelines. Ottawa, ON: CADTH; 2015 Sep 8. | Not a CPG |
| Canadian Agency for Drugs and Technologies in Health. Antidepressants in rlderly patients with major and minor depression: a review of clinical effectiveness and guidelines. Ottawa, ON: CADTH; 2015. | Not a CPG |
| Gensichen J, Härter M, Klesse C, Bermejo I, Bschor T, Harfst T, et al. Germany’s national clinical practice guideline (S3) for unipolar depression - What is important for family practice? ZFA. 87 (5):223–30. https://doi.org/10.3238/zfa.2011.0223. . | Not a CPG |
| [Geoffroy PA, Bellivier F. The RANZCP mood disorders guidelines: An easy step-by-step toolbox for daily practice. Aust N Z J Psychiatry. 2016 Oct;50(10):1014-5. https://doi.org/10.1177/0004867416667829.](http://www.embase.com/search/) | Not a CPG |
| Gitlin M. The Royal Australian and New Zealand College of Psychiatrists clinical guidelines for mood disorders: Kudos and quarrels. Aust N Z Psychiatry. 2016 Oct;50(10):937-8. https://doi.org/10.1177/0004867416668038. | Not a CPG |
| Grobler, G. An overview of depression treatment guidelines. Abstracts 2nd African College of Neuropsychopharmacology Congress 30–31 July 2016 Stellenbosch, Western Cape, South Africa. Acta Neuropsychiatr. 2016;28(s3):1-15. https://doi.org/10.1017/neu.2016.37 | Not a CPG |
| Heilmann KE, Wagner M, Riedel-Heller S, Maier W, Jessen F. [Treating Late Life Depression with Antidepressants: A Summary of Recommendations in International Guidelines]. Fortschr Neurol Psychiatr. 83(7):381-91. <https://doi.org/10.1055/s-0035-1553315>. German. | Not a CPG |
| Horgan D, Dodd S. Combination antidepressants: use by GPs and psychiatrists. Aust Fam Physician. 2011 Jun;40(6):397–400. | Not a CPG |
| Kasper S. Editorial. Fifth issue of 2013. World J Biol Psychiatry. 2013 Jul;14(5):333. https://doi.org/10.3109/15622975.2013.819703. | Not a CPG |
| Kendall T, Pilling S, Glover N, Taylor C. Guidelines in mental health: National and international perspectives. Int Rev Psychiatry. 2011 Aug;23(4):314-7. doi: https://doi.org/10.3109/09540261.2011.607431. | Not a CPG |
| Kongsuk T. Clinical practice guideline major depressive disorder for general practitioners. Value Health. 2013;16(7): A695. https://doi.org/10.1016/j.jval.2013.08.2091. | Not a CPG |
| Kurian BT, Grannemann B, Trivedi MH. Feasible evidence-based strategies to manage depression in primary care. Curr Psychiatry Rep. 2012;14(4):370-5. https://doi.org/10.1007/s11920-012-0290-y. | Not a CPG |
| Laux G. Update treatment of depression - S3 Guideline, internet-based psychotherapy, antidepressants and driving ability. Nervenheilkunde. 2016;35(10):691-6. <https://doi.org/10.1055/s-0037-1616433>. | Not a CPG |
| Leadholm AKK, Rothschild AJ, Nolen WA, Bech P, Munk-Jorgensen P, Ostergaard SD. The treatment of psychotic depression: is there consensus among guidelines and psychiatrists?. J Affective Disord. 145(2): 214–20. https://doi.org/10.1016/j.jad.2012.07.036. | Not a CPG |
| Malhi G, Oakley-Browne M, Hay P. Clinical practice guidelines project (CPG project) overview. Aust N Z J Psychiatry. 2015;49(Supp 1):30. <https://doi.org/10.1177/0004867415578344> | Not a CPG |
| Manning JS, Jackson WC. Providing guideline-concordant assessment and monitoring for major depression in primary care. J Clin Psychiatry. 2015;76(1):e3. https://doi.org/10.4088/JCP.13013tx7c. | Not a CPG |
| Mathys M, Mitchell BG. Targeting treatment-resistant depression. J Pharm Pract.2011;24(6):520-33. https://doi.org/10.1177/0897190011426972. | Not a CPG |
| Morris DW, Trivedi MH. Measurement-based care for unipolar depression. Curr Psychiatry Rep. 13(6);446-58. https://doi.org/10.1007/s11920-011-0237-8. | Not a CPG |
| Nelson JC. Foreword. CNS Drugs. 2013 May 25;27:3-4. https://doi.org/10.1007/s40263-012-0027-9. | Not a CPG |
| Nutt DJ. Highlights of the international consensus statement on major depressive disorder. J. Clin Psychiatry. 2011 Jun;72(6):e21. https://doi.org/10.4088/JCP.9058tx2c. | Not a CPG |
| Ogasawara K, Ozaki N. [Review of the new treatment guideline for major depressive disorder by the Japanese Society of Mood Disorders]. Brain Nerve. 2012 Oct;64(10):1159-65. Japanese. | Not a CPG |
| [Oldham J. Fine-tuning our treatment strategies. J Psychiatr Pract. 2011;17(3): 157. https://doi.org/10.1097/01.pra.0000398408.13750.8d](https://d.docs.live.net/741604ec9d8d6d64/Clientes_Compartilhados_OFICINA%20DE%20IDEIAS/Clientes%20Avulsos_Oficina_Ideias/Gabriel_Franciele/Oldham%20J.%20Fine-tuning%20our%20treatment%20strategies.%20J%20Psychiatr%20Pract.%202011;17(3):%20157.%20https:/doi.org/10.1097/01.pra.0000398408.13750.8d) | Not a CPG |
| Pai N. Are the Royal Australian and New Zealand College of Psychiatrists clinical practice guidelines for mood disorders meeting the needs of clinicians?. Aust N Z J Psychiatry. 2016 Oct;50(10):1015-6. https://doi.org/10.1177/0004867416667828. | Not a CPG |
| Patkar AA, Pae C-U. Atypical antipsychotic augmentation strategies in the context of guideline-based care for the treatment of major depressive disorder. CNS Drugs, 27(Suppl 1):S29-37. https://doi.org/10.1007/s40263-012-0031-0. | Not a CPG |
| Rawlins M. Ten years of NICE mental health guidelines. Int Rev Psychiatry.23(4):311–3. https://doi.org/10.3109/09540261.2011.606804. | Not a CPG |
| Reisdorf S. Revision of the national health care guidelines on unipolar depression. Med Monatss Pharm. 2016;39(4): 171–2. | Not a CPG |
| Roberge P, Fournier L, Brouillet H, Delorme A, Beaucage C, Cote R, et al. A provincial adaptation of clinical practice guidelines for depression in primary care: a case illustration of the ADAPTE method. J Eval Clin Pract. 2015;21(6):1190-8. https://doi.org/10.1111/jep.12404. | Not a CPG |
| Schuklenk U, van de Vathorst S. Treatment-resistant major depressive disorder and assisted dying. J Med Ethics. 2015;41(8), 577–83. https://doi.org/10.1136/medethics-2014-102458. | Not a CPG |
| Schulte-Korne G, Krick K. (2014). In reply. Dtsch Arztebl Int 2014;111(18):330. https://doi.org/10.3238/arztebl.2014.0330c. | Not a CPG |
| Tomba E, Fava GA. Treatment selection in depression: The role of clinical judgment. Psychiat Clin North Am. 2012; 35(1):87–98. https://doi.org/10.1016/j.psc.2011.11.003. | Not a CPG |
| Treuer T, Liu C-Y, Salazar G, Kongsakon R, Jia F, Habil H, Lee M-S, et al. Use of antidepressants in the treatment of depression in Asia: guidelines, clinical evidence, and experience revisited. [Asia Pac Psychiatry.](https://www.ncbi.nlm.nih.gov/pubmed/23857712) 2013 Dec;5(4):219-30. https://doi.org/10.1111/appy.12090. | Not a CPG |
| Tundo A, Calabrese JR, Proietti L, De Filippis R. Short-term antidepressant treatment of bipolar depression: Are ISBD recommendations useful in clinical practice?. J Affect Disord. 2015 Jan 15;171:155–160. https://doi.org/10.1016/j.jad.2014.09.019. | Not a CPG |
| van-Avendonk M, van Weel-Baumgarten E, van-der-Weele G, Wiersma T, Burgers JS [Summary of the Dutch College of General Practitioners’ practice guideline “Depression”]. Ned Tijdschr Geneeskd. 2012;156(38):A5101. | Not a CPG |
| Vinberg M, Levinsen MF, Kessing LV. [Treatment-resistant depression is treatable]. Ugeskr Laeger. 2011 Feb 28;173(9):651-4. Danish. | Not a CPG |
| Wang HR, Bahk WM, Park YM, Lee HB, Song HR, Jeong JH. Korean medication algorithm for depressive disorder: Comparisons with other treatment guidelines. Psychiatry Investig. 2014 Jan;11(1):1-11. https://doi.org/10.4306/pi.2014.11.1.1. | Not a CPG |
| Won E, Park SC, Han KM, Sung SH, Lee HY, Paik JW, et al. Evidence-based, pharmacological treatment guideline for depression in Korea, revised edition. J Korean Med Sci. 2014 Apr;29:(4):468-84. https://doi.org/10.3346/jkms.2014.29.4.468 | Not a CPG |
| Xiang YT, Hu C, Wang G, Zheng QW, Fang YR, Ungvari GS, et al. Prescribing patterns of antidepressants, antipsychotics and mood stabilizers in bipolar patients misdiagnosed with major depressive disorder in China. Hum Psychopharmacol. 2012 Nov;27(6):626-31. https://doi.org/10.1002/hup.2262. | Not a CPG |
| [Yang J](https://www.ncbi.nlm.nih.gov/pubmed/?term=Yang%20J%5BAuthor%5D&cauthor=true&cauthor_uid=23705908), [Han C](https://www.ncbi.nlm.nih.gov/pubmed/?term=Han%20C%5BAuthor%5D&cauthor=true&cauthor_uid=23705908), [Yoon HK](https://www.ncbi.nlm.nih.gov/pubmed/?term=Yoon%20HK%5BAuthor%5D&cauthor=true&cauthor_uid=23705908), [Pae CU](https://www.ncbi.nlm.nih.gov/pubmed/?term=Pae%20CU%5BAuthor%5D&cauthor=true&cauthor_uid=23705908), [Kim MJ](https://www.ncbi.nlm.nih.gov/pubmed/?term=Kim%20MJ%5BAuthor%5D&cauthor=true&cauthor_uid=23705908), [Park SY](https://www.ncbi.nlm.nih.gov/pubmed/?term=Park%20SY%5BAuthor%5D&cauthor=true&cauthor_uid=23705908), et al. Experiences and barriers to implementation of clinical practice guideline for depression in Korea. BMC Psychiatry. 2013 May 27;13:150. https://doi.org/10.1186/1471-244X-13-150. | Not a CPG |
| Zimmerman M. Symptom severity and guideline-based treatment recommendations for depressed patients: implications of DSM-5’s potential recommendation of the PHQ-9 as the measure of choice for depression severity. Psychother Psychosom. 2012;81(6):329-32. https://doi.org/10.1159/000342262. | Not a CPG |
| Zimmerman M, Martinez JH, Friedman M, Boerescu DA, Attiullah N, Toba C. How can we use depression severity to guide treatment selection when measures of depression categorize patients differently?. J Clin Psychiatry. 2012 Oct;73(10):1287-91. https://doi.org/10.4088/JCP.12m07775. | Not a CPG |
| Evidence-Based Clinical Practice Guidelines for depression in Adults on Traditional Korean Medicine, 2016. | Not found |
| S3-Leitlinie/Nationale Versorgungs Leitlinie Unipolare Depression. 2015. German. | Language |
| [Depression. Adapted evidence-based guideline. Ucrania]. 2014 - Депресія. Адаптованаклінічнанастанова. Ukrainian. | Language |
| [Depressive disorder. Depressie (M44)]. 2012. Dutch. | Language |
| Herrmann M, Gensichen J. [Guidelines in general practice: The example depression]. Med Welt. 2015;66:119-24. | Language |
| Kuroki T, Tanaka T. [Threshold of Application of Antidepressant Drugs for Treatment of Depressive Disorder]. Seishin Shinkeigaku Zasshi. 2015;117(4):269-76. [Japanese]. | Language |
| Semba J. [Proper use of novel antidepressants in psychiatric clinical practice]. Seishin Shinkeigaku Zasshi. 2014;116(2):138-43. [Japanese]. | Language |
| Yamada K. [Evidence of treatment for depressive episodes of bipolar disorder]. Seishin Shinkeigaku Zasshi. 2011;113(9):873-9. [Japanese]. | Language |
| Elliott LN, Merlo GB, Campbell SA, Norris SK, organizers. Clinical Practice Guidelines: Depression and related disorders – anxiety, bipolar disorder and puerperal psychosis – in the perinatal period. [Melbourne]: Beyondblue; 2009. | Focus on special groups |
| [Clinical Practice Guidelines: Depression in Adolescents and Young Adults. [Melbourne]: NHMCRC, [2015]](https://d.docs.live.net/741604ec9d8d6d64/Clientes_Compartilhados_OFICINA%20DE%20IDEIAS/Clientes%20Avulsos_Oficina_Ideias/Gabriel_Franciele/Clinical%20Practice%20Guidelines:%20Depression%20in%20Adolescents%20and%20Young%20Adults.%20%5bMelbourne%5d:%20NHMCRC,%20%5b2015%5d.). | Focus on special groups |
| Rayner L, Price A, Hotopf M, Higginson IJ. The development of evidence-based European guidelines on the management of depression in palliative cancer care. EurJ Cancer. 2011; 47(5):702-12. https://doi.org/10.1016/j.ejca.2010.11.027. | Focus on special groups |
| World Health Organization. Pharmacological interventions (antidepressants) for 433 people with dementia who have associated depression [cited 2017 August 1]. [n.p.]: WHO; 2015. Available from: https://www.who.int/mental_health/mhgap/evidence/dementia/q4/en/. | Focus on special groups |
| Colquhoun DM, Bunker SJ, Clarke DM, Glozier N, Hare DL, Hickie IB, et al. Screening, referral and treatment for depression in patients with coronary heart disease. Med J Aust. 2013 May 20;198(9):483-4. https://doi.org/10.5694/mja13.10153. | Focus on special groups |
| Galician Health Technology Assessment Agency. Clinical practice guideline on the management of major depression in adults. [Santiago de Compostela]: Galician Health Technology Assessment Agency, 2008. | Outdated |
| National Collaborating Centre for Mental Health (UK). Depression. The treatment and management of depression in adults. Leicester (UK): British Psychological Society; 2010. | Outdated |
| Lam RW, Parikh SV, Michalak EE, Dewa CS, Kennedy SH. Canadian Network for Mood and Anxiety Treatments (CANMAT) consensus recommendations for functional outcomes in major depressive disorder. Ann Clin Psychiatry. 2015 May;27(2):142-9. | Outdated |
| Agency for Healthcare Research and Quality. Detecting depression in older adults with dementia. ... University of Michigan Health System. [Michigan]: [University of Michigan]; [s.d]. | Outdated |
| Adult depression in primary care. Bloomington, MN: ICSI; 2013. | Outdated |
| Galletly C1, Castle D2, Dark F3, Humberstone V4, Jablensky A5, Killackey E. Royal Australian and New Zealand college of psychiatrists clinical practice guideline project and clinical practice guidelines for anxiety disorders, mood disorders, schizophrenia and related disorders. Aust N Z J Psychiatry. 2016 May;50(5):410-72. https://doi.org/10.1177/0004867416641195. | Outdated |
| Belgian Health Care Knowledge Centre. The long-term efficacy of psychotherapy, alone or in combination with antidepressants, in the treatment of adult major depression. 2014 Oct 2 [cited 2017 June 30]. Available from: https://kce.fgov.be/publication/report/the-long-term-efficacy-of-psychotherapy-alone-or-in-combination-with-antidepressa#.WL2nUH-53sc. | Focus on psychotherapy |
