# Supplementary material for: Pharmacological treatment of depression: A systematic review comparing clinical practice guideline recommendations
Source: PLoS One. 2020 Apr 21;15(4):e0231700. doi: 10.1371/journal.pone.0231700 (PMC7173786; doi:10.1371/journal.pone.0231700)
Supplement: S3 Appendix — (DOCX) [file pone.0231700.s003.docx]

**S3 Appendix. Quality assessment of the included clinical practice guidelines**

The quality of the selected CPGs was assessed using AGREE II. Per AGREE II, each question is scored on a seven-point Likert scale: 1 (“strongly disagree”) to 7 (“strongly agree”). Three assessors, trained to use AGREE II, as described in a previous study [1], performed the CPG analyses to increase the reliability of results as established by the Agree Next Steps Consortium. They independently analysed each CPG, including supplementary documents, and gave their scores on the AGREE II Platform. Thus, each question of the AGREE II to assess the quality of the CPGs received 3 grades (i.e., one from each of the 3 assessors). These grades were considered divergent when a difference of two or more points was observed. All divergences were resolved by attaining a consensus among the assessors. In the absence of a consensus among the three assessors, a senior investigator was requested to participate in the discussion and establish the grade. Domain 3, from the AGREE II, was adopted to establish CPG quality. This domain evaluates the methodological rigor during the prosses of CPG development, and it is the most relevant domain for the reliability of recommendations [2-3].
