# Supplementary material for: Pharmacological treatment of depression: A systematic review comparing clinical practice guideline recommendations
Source: PLoS One. 2020 Apr 21;15(4):e0231700. doi: 10.1371/journal.pone.0231700 (PMC7173786; doi:10.1371/journal.pone.0231700)
Supplement: S1 Table — (DOCX) [file pone.0231700.s004.docx]

**S1 Table. Recommendations for the treatment of depression, extracted from clinical practice guidelines, listed for elaborating the synthesis published between January 2011 and April 2019: indications and strategies of pharmacological treatment**

| **Recommendations** | **Clinical practice guidelines** | **Evidence classification scale** | **Strength of recommendation** | **Level of evidence** |  |
| --- | --- | --- | --- | --- | --- |
| **RISK OF SUICIDE** | | | | |  |
| Patients on antidepressants and presenting as a suicide risk should be monitor  ed regularly. | [4] | GRADE | Strong | _ |  |
|  | [5] | GRADE | _ | _ |  |
| The risk of overdose toxicity is higher in patients using tricyclic antidepressants. NICE, 2016 [5]: except lofrepramine | [4] | GRADE | Strong | _ |  |
|  | [5] | GRADE | _ | _ |  |
| Overdose toxicity can occur with all antidepressants but is more with dual-action antidepressants. | [4] | GRADE | Strong | _ |  |
| Venlafaxine (a dual-action antidepressant) is associated with a higher risk of death by overdose. | [5] | GRADE | _ | _ |  |
| The choice of the type of intervention must be influenced by the response to the previous treatment for depression. | [5] | GRADE | _ | _ |  |
| Pharmacotherapy combined with psychotherapy is a way to increase the intensity of treatment for patients who are at risk of suicide. | [6] | MODIFIED GRADE | Recommended with moderate clinical safety | _ |  |
| The need for hospitalization should be considered in patients at risk of suicide. | [6] | MODIFIED GRADE | Recommended with significant clinical safety | _ |  |
| **ACUTE PHASE TREATMENT** | | | | |  |
| **GOAL** | | | | |  |
| Reach remission of the depressive episode and return to the patient’s base level | [6] | MODIFIED GRADE | Recommended with significant clinical safety | _ |  |
| **INDICATION FOR PHARMACOLOGICAL TREATMENT** | | | | |  |
| Antidepressants should not be used routinely to treat mild depression. | [7] | MODIFIED GRADE | Highly recommended, based on good-quality studies | Systematic reviews, meta-analyses, randomized trials, and health technology assessment reports |  |
|  | [5] | GRADE | _ | _ |  |
|  | [4] | GRADE | Strong | _ |  |
|  | [8] | OTHER | _ | _ |  |
| Antidepressants can be used to treat patients with persistent mild depression or who have a history of moderate/serious depression.^*^  ^*^[5] specifies that this is depression that lasts for at least 2 years. | [4] | GRADE | Strong | _ |  |
|  | [5] | GRADE | _ | _ |  |
| Antidepressants are indicated for the treatment of severe depression. | [6] | MODIFIED GRADE | Recommended with significant clinical safety | _ |  |
|  | [4] | GRADE | Strong | _ |  |
| Antidepressants should be used in serious depression unless electroconvulsive therapy is planned. | [6] | MODIFIED GRADE | Recommended with significant clinical safety | _ |  |
| Options for treatment are pharmacotherapy, psychotherapy, a combination of pharmacotherapy and psychotherapy, electroconvulsive therapy, transcranial magnetic stimulation, and phototherapy. | [6] | MODIFIED GRADE | _ | _ |  |
| When the reaction to loss is too extensive or there is functional impairment, psychotherapy, or antidepressants should be used. | [6] | MODIFIED GRADE | Recommended with significant clinical safety | _ |  |
| Antidepressants can be a treatment choice for mild-to-moderate depression. | [6] | MODIFIED GRADE | Recommended with significant clinical safety | _ |  |
| Antidepressants can be a treatment choice for moderate depression. | [4] | GRADE | Strong | _ |  |
|  | [6] | MODIFIED GRADE | Recommended with significant clinical safety | _ |  |
| **INDICATION OF A COMBINATION PHARMACOTHERAPY—PSYCHOTHERAPY** | | | | |  |
| Consider combining antidepressant and psychotherapy whenever possible | [9] | GRADE | Weak | Moderate |  |
| If combined treatment is not possible (pharmacotherapy + psychotherapy), initiate treatment with pharmacotherapy in serious depression. | [9] | GRADE | Weak | Moderate |  |
| For patients with moderate depression consider a combination of antidepressants and psychotherapy.^*^  ^*^[5] suggests cognitive-behavioural therapy or interpersonal psychotherapy | [4] | GRADE | Strong | _ |  |
|  | [5] | GRADE | Weak | _ |  |
|  | [6] | MODIFIED GRADE | Recommended with significant clinical safety | _ |  |
| For patients with serious depression, use psychotherapy in conjunction with antidepressants. | [7] | MODIFIED  GRADE | Highly recommended, based on good-quality studies | Systematic reviews, meta-analyses, randomized trials, and health technology assessment reports |  |
|  | [5] | GRADE | _ | _ |  |
|  | [4] | GRADE | Strong | _ |  |
|  | [6] | MODIFIED  GRADE | Recommended with significant clinical safety | _ |  |
| For patients with mild depression who have psychosocial or interpersonal issues, intrapsychic conflicts, or axis II disorders, a combination of antidepressants and psychotherapy can be useful. | [6] | MODIFIED GRADE | Recommended with moderate clinical safety | _ |  |
| **CHOICE OF MEDICATION** | | | | |  |
| Selective serotonin reuptake inhibitors are recommended as first-line options for antidepressant treatment. | [7] | MODIFIED GRADE | Highly recommended, based on good-quality studies | Systematic reviews, meta-analyses, randomized trials, and health technology assessment reports |  |
|  | [5] | GRADE | _ | _ |  |
|  | [4] | GRADE | Strong | _ |  |
|  | [9] | GRADE | _ | _ |  |
|  | [6] | MODIFIED GRADE | Recommended with significant clinical safety | _ |  |
|  | [8] | OTHER | _ | Level 1 |  |
| Tricyclic antidepressants are not recommended as first-line drugs owing to the associated adverse events. | [7] | MODIFIED GRADE | Highly recommended, based on good-quality studies | Systematic reviews, meta-analyses, randomized trials, and health technology assessment reports |  |
|  | [9] | GRADE | _ | _ |  |
| Amitriptyline (a tricyclic antidepressant) can be considered as a first-line drug option. Exception: elderly patients who present contraindications to tricyclic antidepressants. | [4] | GRADE | Strong | _ |  |
| For patients with contraindications for the use of tricyclic antidepressants, consider fluoxetine, sertraline, or mirtazapine as first-line options. | [4] | GRADE | Strong | _ |  |
| Bupropiona can be considered a first-line drug | [9] | GRADE | _ | _ |  |
|  | [8] | OTHER | _ | Level 1 |  |
|  | [6] | MODIFIED GRADE | Recommended with significant clinical safety | _ |  |
| Venlafaxine, duloxetine, desvenlafaxine, and vortioxetine can be considered as first-line drugs. | [9] | GRADE | _ | _ |  |
|  | [8] | OTHER | _ | Level 1 |  |
| Mirtazapine can be considered a first-line drug option. | [4] | GRADE | Strong | _ |  |
|  | [9] | GRADE | _ | _ |  |
|  | [8] | OTHER | _ | Level 1 |  |
|  | [6] | MODIFIED GRADE | Recommended with significant clinical safety | _ |  |
| Levomilnacipran and vilazodone can be considered as first-line drug options. | [ 9] | GRADE | _ | _ |  |
| Agomelatine, citalopram, escitalopram, fluoxetine, fluvoxamine, mianserin, milnacipran, paroxetine, and sertraline | [8] | OTHER | _ | Level 1 |  |
| **CONTINUATION PHASE** | | | | |  |
| The choice of treatment to reduce the risk of relapse (continuation phase) and recurrence should be discussed with the patient after considering prior treatment, discontinuation symptoms, and patient preferences. | [5] | GRADE | _ | _ |  |
| Pharmacological treatment should be maintained^*^ for at least 6 months after remission of symptoms.  ^*^stipulates a period of 6–12 months [4,7].  ^*^mentions 6–9 months [8]. | [7] | MODIFIED GRADE | Recommended with moderate clinical safety | Systematic reviews, meta-analyses, randomized trials, and health technology assessment reports |  |
|  | [5] | GRADE | _ | _ |  |
|  | [4] | GRADE | Strong | _ |  |
|  | [8] | OTHER | _ | _ |  |
| Pharmacological treatment should extend from 4 to 9 months after remission of symptoms in patients who have undergone successful treatment with antidepressants. | [6] | MODIFIED GRADE | Recommended with significant clinical safety | _ |  |
| Explain to the patient that continuing the use of medication after remission considerably reduces the risk of relapse and that antidepressants are not associated with addiction. | [5] | GRADE | _ | _ |  |
| The same dosage of tricyclic antidepressant, although low, can be continued for patients who had a clear response, maintaining careful monitoring. | [5] | GRADE | _ | _ |  |
| The same dosage used during the acute phase should generally be used in the continuation phase. | [6] | MODIFIED GRADE | Recommended with moderate clinical safety | _ |  |
| People who had several episodes of depression and who responded to treatment with an antidepressant combined with augmentation agents should continue this combination after remission if they find that the adverse events are tolerable and acceptable. | [5] | GRADE | _ | _ |  |
| For patients who received electroconvulsive therapy, the continuation treatment should be lithium and nortriptyline. | [6] | MODIFIED GRADE | Recommended with significant clinical safety | _ |  |
| **MAINTENANCE PHASE** | | | | |  |
| For continued treatment with antidepressants, consider the number^*^ of prior episodes of depression.  ^*^[ 7] specifies that three or more depressive episodes are necessary. | [7] | MODIFIED GRADE | Highly recommended, based on good-quality | Systematic reviews, meta-analyses, randomized trials, and health technology assessment reports |  |
|  | [5] | GRADE | _ | _ |  |
|  | [4] | GRADE | Weak | _ |  |
|  | [6] | MODIFIED GRADE | Recommended with significant clinical safety | _ |  |
|  | [8] | OTHER | _ | _ |  |
| For continued treatment with antidepressants, consider the presence of residual symptoms, concomitant physical health issues, and psychosocial difficulties. | [4] | GRADE | Weak | _ |  |
|  | [5] | GRADE | _ | _ |  |
|  | [6] | MODIFIED GRADE | Recommended with significant clinical safety | _ |  |
|  | [8] | OTHER | _ | Level 3 e Level 4 |  |
| The severity of depressive symptoms should also be considered while making decisions regarding the maintenance phase. | [4] | GRADE | Weak | _ |  |
|  | [5] | GRADE | _ | _ |  |
|  | [8] | MODIFIED GRADE | Recommended with moderate clinical safety | _ |  |
| For people with depression who are at a significant risk of relapse or have a history of recurrent depression, explain the treatment options to reduce recurrence, including continuous medication, augmentation of the medication or psychological treatment (cognitive-behavioural therapy). | [5] | GRADE | _ | _ |  |
| Continue with antidepressants for 2 years if the consequences of relapses can be serious, such as suicide attempts and inability to work. | [5] | GRADE | _ | _ |  |
| We should extend the treatment for 2 years or more in the presence of risk factors. | [4] | GRADE | Weak | _ |  |
|  | [8] | OTHER | _ | Level 3 Level 4 |  |
|  | [5] | GRADE | _ | _ |  |
| Maintain the dosage of the drug that was effective if the adverse events are tolerable. | [5] | GRADE | _ | _ |  |
| During the maintenance phase, an antidepressant drug that produced remission of symptoms during the acute phase and persistence of remission during the continuation phase should be administered in full therapeutic dosage. | [6] | MODIFIED GRADE | Recommended with moderate clinical safety | _ |  |
| When deciding whether to continue the maintenance treatment beyond 2 years, consider the age. | [5] | GRADE | _ | _ |  |
| A family history of mood disorders and an early age increase the possibility of recurrent episodes and support the need for maintenance treatment. | [6] | MODIFIED GRADE | Recommended with moderate clinical safety | _ |  |
| The frequency of depressive symptoms and persistence contribute to decisions regarding the maintenance phase. | [6] | MODIFIED GRADE | Recommended with moderate clinical safety | _ |  |
| The patient’s preference, the type of treatment received, and the adverse events during the continuation phase should all be considered while making maintenance phase decisions. | [6] | MODIFIED GRADE | Recommended with moderate clinical safety | _ |  |
| For patients with chronic depression and concomitant psychiatric treatment, indefinite maintenance treatment is necessary. | [6] | MODIFIED GRADE | Recommended with significant clinical safety | _ |  |
| **DISCONTINUATION OF TREATMENT** | | | | |  |
| Before discontinuing the treatment, patients should be informed regarding the risk of relapse. | [6] | MODIFIED GRADE | Recommended with significant clinical safety | _ |  |
| Discontinue the antidepressant gradually^*^to avoid discontinuation symptoms.  ^*^NICE, 2016 [5] mentions 4 weeks. | [4] | GRADE | Strong | _ |  |
|  | [5] | GRADE | _ | _ |  |
|  | [6] | MODIFIED GRADE | Recommended with significant clinical safety | _ |  |
| Paroxetine and venlafaxine have a shorter half-life and require a more gradual dosage reduction to minimize discontinuation symptoms. | [4] | GRADE | Strong | _ |  |
|  | [5] | GRADE | _ | _ |  |
| Inform the patients that discontinuation symptoms can occur with interruption of treatment or in the event of forgetting dosages. | [6] | MODIFIED GRADE | Recommended with significant clinical safety | _ |  |
|  | [5] | GRADE | _ | _ |  |
| When discontinuation symptoms occur take necessary action as follows:  - If mild, monitor and reassure the patient  - If severe, consider reintroducing the antidepressant at the dosage that was effective (or another antidepressant of the same class with a longer half-life) and gradually reduce the dosage while monitoring the symptoms. | [5] | GRADE | _ | _ |  |
| If serious discontinuation symptoms occur, an antidepressant with a longer half-life can be administered to minimize these effects. | [6] | MODIFIED GRADE | Recommended with moderate clinical safety | _ |  |
|  | [5] | GRADE | _ | _ |  |
| If there is no improvement in the first month, treatment (psychotherapy and/or pharmacotherapy) should be discontinued. | [6] | MODIFIED GRADE | Recommended with significant clinical safety | _ |  |
