# Supplementary material for: Pharmacological treatment of depression: A systematic review comparing clinical practice guideline recommendations
Source: PLoS One. 2020 Apr 21;15(4):e0231700. doi: 10.1371/journal.pone.0231700 (PMC7173786; doi:10.1371/journal.pone.0231700)
Supplement: S2 Table — (DOCX) [file pone.0231700.s005.docx]

**S2 Table. Recommendations for the treatment of depression, extracted from clinical practice guidelines, listed for elaborating the synthesis published between January 2011 and April 2019: patients who did not respond or partially responded**

| **Recommendations** | **Clinical practice guidelines** | **Evidence classification scale** | **Recommendation strength** | **Level of evidence** |
| --- | --- | --- | --- | --- |
| **PATIENTS WHO DID NOT RESPOND OR SHOWED PARTIAL RESPONSE** | | | | |
| **ANALYSIS OF CONTRIBUTING FACTORS** | | | | |
| Check adherence to treatment and prescribed dosage in patients who did not respond satisfactorily to interventions.^*^  ^*^[5] mentions a 2–4-week period assessing the response to interventions. | [6] | MODIFIED GRADE | Recommended with significant clinical safety | _ |
|  | [5] | GRADE | _ | _ |
| The physician should assess all patients for the presence of factors that indicate a bad response to adjust follow-up and alter the treatment for depression.^*^  ^*^[4]: Disorders that are predictors of a bad response are personality disorders, suicidal intent, comorbidities, high body mass index, unemployment, physical pain, and chronic depression. | [4] | GRADE | Weak | _ |
| Check duration, frequency, and dosage of the drug administered for assessing the adequacy of the intervention. | [6] | MODIFIED GRADE | Recommended with significant clinical safety | _ |
| If the symptoms do not improve in 4–8 weeks, re-evaluate—diagnosis, comorbidity complications, psychosocial factors, therapeutic alliance, and adherence—and modify treatment. | [6] | MODIFIED GRADE | Recommended with significant clinical safety | _ |
| If there is no improvement in 2–4 weeks, change the treatment plan (increase dosage or replace drug). | [8] | OTHER | _ | _ |
| While analysing pharmaceutical treatment for a person with depression whose symptoms did not adequately respond to the initial pharmacological interventions, check for adverse effects. | [6] | MODIFIED GRADE | Recommended with significant clinical safety | _ |
|  | [5] | GRADE | _ | _ |
| **TREATMENT STRATEGIES** | | | | |
| For patients who did not respond to drugs or psychological interventions, combine psychotherapy with drugs. | [6] | MODIFIED GRADE | Recommended with moderate clinical safety | _ |
|  | [5] | GRADE | _ | _ |
| For people with mild depression who did not benefit from low-intensity psychosocial intervention, provide an antidepressant or a high-intensity psychological intervention. | [5] | GRADE | _ | _ |
| While reviewing the pharmacological treatment, be aware that using a single antidepressant rather than a combination of drugs or augmentation agents is usually linked to a lower risk of adverse effects. | [5] | GRADE | _ | _ |
| In patients who did not respond to the initial pharmacological interventions in 3–4 weeks, consider reintroducing prior treatments that showed high adherence by the patient, and increase the level of support (in person and over the phone). | [5] | GRADE | _ | _ |
| If during the third week of treatment the patient’s symptoms have not improved despite proper adherence, increase the dosage or replace the drug. | [4] | GRADE | Strong | _ |
| **ADJUSTING THE DOSAGE OF DRUGS** | | | | |
| Once antidepressant drugs have been initiated, gradual dosage adjustment should depend on the patient’s age, the presence of comorbidities, concomitant drug treatment, and adverse effects caused by the drugs. | [6] | MODIFIED GRADE | Recommended with significant clinical safety | _ |
|  | [5] | GRADE | _ | _ |
| The authors recommended gradually increasing the dosages of selective serotonin reuptake inhibitors in patients who do not respond to lower dosages. | [9] | GRADE | _ | _ |
| Increasing the dosage of tricyclics or monoamine oxidase inhibitors may improve the response. | [9] | GRADE | _ | _ |
| For patients who are taking tricyclic antidepressants, monitoring the blood levels of the drugs can help determine whether additional dosage adjustments are required. | [6] | MODIFIED GRADE | Recommended with significant clinical safety | _ |
| Consider increasing the dosage of the prescribed drugs if the response is inadequate.^*^  ^*^[5] recommends this if the response is minimal or absent in a 3–4-week period. | [4] | GRADE | Strong | _ |
|  | [5] | GRADE | _ | _ |
|  | [6] | MODIFIED GRADE | Recommended with moderate clinical safety | _ |
|  | [8] | OTHER | _ | _ |
|  | [9] | GRADE | _ | _ |
|  | [7] | MODIFIED GRADE | Highly recommended  Based on good-quality studies | Systematic reviews, meta-analyses, randomized trials, and health technology assessment reports |
| For patients who require changes in the treatment plan, optimizing the dosage is a possibility.^*^  ^*^[6,7] recommend this only if the adverse effects are tolerable. | [7] | MODIFIED GRADE | Highly recommended  Based on good-quality studies | Systematic reviews, meta-analyses, randomized trials, and health technology assessment reports |
|  | [6] | MODIFIED GRADE | Recommended with moderate clinical safety | _ |
| If drugs are prescribed, the psychiatrist must determine whether pharmacokinetic factors suggest the need to adjust the dosages of the drugs. | [6] | MODIFIED GRADE | Recommended with significant clinical safety | _ |
| If drugs are prescribed, the psychiatrist must determine whether pharmacodynamic factors suggest the need to adjust the dosages of the drugs. | [6] | MODIFIED GRADE | Can be recommended according to individual circumstances | _ |
| **CHANGING DRUGS** | | | | |
| In patients who did not respond to the initial pharmacological interventions, consider changing the antidepressant. | [7] | MODIFIED GRADE | Highly recommended  Based on good-quality studies | Systematic reviews, meta-analyses, randomized trials, and health technology assessment reports |
|  | [5] | GRADE | _ | _ |
|  | [4] | GRADE | Strong | _ |
|  | [6] | MODIFIED GRADE | _ | _ |
|  | [9] | GRADE | _ | _ |
|  | [8] | OTHER | _ | _ |
| Consider switching to another antidepressant under the following circumstances:  ^*^The current medication is the first antidepressant tried.  ^*^There are ill tolerated adverse events from the initial antidepressant.  ^*^There is no response (< 4% of improvement) for the initial antidepressant.  ^*^There is more time to wait for a response (less serious and less functional deficit).  ^*^The patient prefers to change to another antidepressant. | [8] | OTHER | _ | Level 3 |
| If the depression does not improve within 4 weeks, continue treatment for a further of 2 weeks and consider changing the antidepressant. | [5] | GRADE | _ | _ |
| If the depression shows improvement in 4 weeks, continue treatment for 2–4 weeks. Consider changing the antidepressant if the response is still inadequate, there are adverse events, or the person prefers to replace the treatment. | [5] | GRADE | _ | _ |
| The antidepressants can be switched within the same class or between different classes. | [6] | MODIFIED GRADE | Recommended with moderate clinical safety | _ |
| For patients that do not respond to the selective serotonin reuptake inhibitor, replace with noradrenaline selective inhibitor. | [6] | MODIFIED GRADE | Recommended with moderate clinical safety | _ |
| While changing the antidepressant, be aware that the evidence of the relative advantage of changing within or between classes is weak. Consider changing to a different selective serotonin reuptake inhibitor initially or a better tolerated antidepressant from a more recent generation; subsequently, use an antidepressant from a different pharmacological class that can be less well tolerated; for example, venlafaxine, a tricyclic antidepressant, or monoamine oxidase inhibitor. | [4] | GRADE | Weak | _ |
|  | [5] | GRADE | _ | _ |
| Consider possible drug interactions while changing antidepressants. | [4] | GRADE | Strong | _ |
|  | [5] | GRADE | _ | _ |
| While changing to an irreversible monoamine oxidase inhibitor, do not routinely prescribe other antidepressants for a time^*^to avoid drug interactions.  ^*^[4,5] specify that the time mentioned is 2 weeks. | [4] | GRADE | Strong | _ |
|  | [5] | GRADE | _ | _ |
|  | [6] | MODIFIED GRADE | Recommended with significant clinical safety | _ |
| Be particularly careful when changing from fluoxetine to other antidepressants because it has a long half-life. | [4] | GRADE | Strong | _ |
|  | [5] | GRADE | _ | _ |
| While changing fluoxetine and paroxetine to a tricyclic antidepressant, start with lower dosages because these selective serotonin reuptake inhibitors inhibit the metabolism of tricyclic antidepressants. | [4] | GRADE | Strong | _ |
|  | [5] | GRADE | _ | _ |
| Be careful when changing to a new serotoninergic antidepressant or monoamine oxidase inhibitors owing to the risk of serotonin syndrome. | [4] | GRADE | Strong | _ |
|  | [5] | GRADE | _ | _ |
| If it is necessary to change the antidepressant used as second-line of treatment, first-line alternatives that have not been prescribed, such as fluoxetine or sertraline (selective serotonin reuptake inhibitors), amitriptyline (a tricyclic antidepressant) or mirtazapine (a specific noradrenergic and serotoninergic antidepressant) should be used. | [4] | GRADE | Strong | _ |
| In patients who do not respond to other treatments, an additional option is to switch to a non-selective monoamine oxidase inhibitor.^*^  ^*^[4] recommends monoamine oxidase inhibitors as a fourth-line treatment. | [4] | GRADE | Weak | _ |
|  | [6] | MODIFIED GRADE | Recommended with moderate clinical safety | _ |
|  | [9] | GRADE | _ | _ |
| Transdermal selegiline, a monoamine oxidase inhibitor that is relatively selective and has less dietary and pharmaceutical restrictions, can be considered. | [6] | MODIFIED GRADE | Recommended with moderate clinical safety | _ |
| For patients who showed minimal improvement or experienced considerable adverse events owing to the medication, other options include changing to another antidepressant that does not inhibit monoamine oxidase. | [6] | MODIFIED GRADE | Recommended with significant clinical safety | _ |
|  |  |  |  |  |
| Recommended second-line agents include tricyclic antidepressants, quetiapine and trazodone, moclobemide, selegiline, levomilnacipran, and vilazodone. | [8] | OTHER | _ | Level 1 |
| Amitripline can be a second-line treatment option^*^  ^*^[4] recommends amitriptiline as a first- or second-line option (if the drug has not been prescribed as first line, and drug switching is necessary). | [4] | GRADE | Strong | _ |
|  | [8] | OTHER | _ | Level 1 |
| Recommended second-line agents include tricyclic antidepressants, quetiapine and trazodone, moclobemide, selegiline, levomilnacipran, and vilazodone. | [8] | OTHER | _ | Level 1 |
| The third-line treatment includes imipramine, clomipramine, paroxetine, escitalopram, citalopram, fluvoxamine, venlafaxine, duloxetine, desvenlafaxine, trazodone, and bupropion. | [4] | GRADE | Strong | _ |
| Monoamine oxidase inhibitors and reboxetine can be considered third-line treatment agents. | [8] | OTHER | _ | Level 1 |
| Phenelzine, tranylcypromine and reboxetine can be considered third-line treatment agents. | [8] | OTHER | _ | Level 1 |
| **DRUG COMBINATION^1^ OR AUGMENTATION EFFECT^2^**  ^1^Combination is when two antidepressants are used in conjunction [ 6]  ^2^Augmentation is when one antidepressant is used together with a non-antidepressant drug [ 6] | | | | |
| Consider an adjuvant medication under the following circumstances:  ^*^There were two or more previous trials with antidepressants.  ^*^The initial antidepressant is well tolerated.  ^*^There is a partial response (over 4% of improvement) to the initial antidepressant.  ^*^There are specific residual symptoms or adverse events for the initial antidepressant that can be tackled.  ^*^There is less time to wait for a response (more serious and more functional deficit).  ^*^The patient prefers to add another medication. | [8] | OTHER | _ | Level 3 |
| It is not recommended to routinely use antidepressant augmentation with benzodiazepines, buspirone, carbamazepine, lamotrigine, valproate, or clonidine. | [4] | GRADE | Weak | _ |
| Antipsychotics can be used as augmentation agents. | [4] | GRADE | Weak | _ |
|  | [9] | GRADE | _ | _ |
|  | [5] | GRADE | _ | _ |
|  | [6] | MODIFIED GRADE | Recommended with significant clinical safety | _ |
|  | [8] | OTHER | _ | _ |
| When prescribing atypical antipsychotics as augmentation agents, their efficiency and safety should be reassessed often. | [9] | GRADE | _ | _ |
| Aripiprazol and quetiapine can be considered adjuvant agents.^*^  ^*^[8] mentions that these are first-line adjuvant treatments. | [4] | GRADE | Weak | _ |
|  | [9] | GRADE | _ | _ |
|  | [5] | GRADE | _ | _ |
|  | [8] | OTHER | _ | _ |
| Antidepressants can also be used as augmentation agents | [7] | MODIFIED  GRADE | Highly recommended  Based on good-quality studies | Systematic reviews, meta-analyses, randomized trials, and health technology assessment reports |
|  | [4] | GRADE | Recommended with significant clinical safety | _ |
| It is possible to augmentation the treatment using antidepressants other than monoamine oxidase inhibitors. | [6] | MODIFIED  GRADE | Recommended with moderate clinical safety | _ |
|  | [4] | GRADE | Weak | _ |
| Olanzapine can be considered an adjuvant treatment.^*^  ^*^[5] uses the term “augmentation treatment.” | [5] | GRADE | _ | _ |
|  | [9] | GRADE | _ | _ |
|  | [8] | OTHER | _ | _ |
| Mirtazapine (an antidepressant) can be used as adjuvant agent.^*^  ^*^[5] uses the term “augmentation treatment.” | [9] | GRADE | _ | _ |
|  | [8] | OTHER | _ | _ |
|  | [5] | GRADE | _ | _ |
| Risperidone (an antipsychotic) can be used as adjuvant agent.^*^  ^*^[8] mentions that these are first-line adjuvant treatments. | [4] | GRADE | Weak | _ |
|  | [8] | OTHER | _ | Level 1 |
|  | [5] | GRADE | _ | _ |
| Lithium can be considered as an augmentation agent^*^  ^*^[8] mentions lithium as a second-line adjuvant treatment agent and does not use the term “augmentation.” | [7] | MODIFIED GRADE | Highly recommended  Based on good-quality studies | Systematic reviews, meta-analyses, randomized trials, and health technology assessment reports |
|  | [4] | GRADE | Weak | _ |
|  | [8] | OTHER | _ | Level 2 |
|  | [5] | GRADE | _ | _ |
|  | [6] | MODIFIED GRADE | Recommended with moderate clinical safety | _ |
| Lithium should not be used as single agent to avoid recurrence. | [5] | GRADE | _ | _ |
| If a drug must be discontinued, select the augmentation agent. | [5] | GRADE | _ | _ |
| In primary care, the combination of drugs should only be done after referral to a psychiatrist. | [5] | GRADE | _ | _ |
| Use only combinations/associations of drugs known to be safe. | [5] | GRADE | _ | _ |
| Explain the logic of any combination to the person with depression. | [5] | GRADE | _ | _ |
| While combining drugs, carefully monitor adverse events and be aware that this generally causes an increase in the effects. | [5] | GRADE | _ | _ |
| Be familiar with primary evidence and consider obtaining a second opinion when using uncommon combinations of drugs. | [5] | GRADE | _ | _ |
| Document the logic for the drug combination selected. | [5] | GRADE | _ | _ |
| Do not routinely use augmentation of an antidepressant with a benzodiazepine for longer than 2 weeks because there is a risk for dependency. | [5] | GRADE | _ | _ |
| It is not recommended to routinely augment antidepressants with benzodiazepines, buspirone, carbamazepine, lamotrigine, and valproate. | [5] | GRADE | _ | _ |
|  | [4] | GRADE | Weak | _ |
| Benzodiazepines can be used as adjuvant treatment with antidepressants in patients with depression and concomitant anxiety. | [6] | MODIFIED GRADE | Recommended with moderate clinical safety | _ |
| Do not routinely augmentation an antidepressant with clonidine. | [4] | GRADE | Weak | _ |
| Augmentation strategies include using an anticonvulsant, omega-3 fatty acids, folate, or psychoactive medication, including modafinil. | [6] | MODIFIED GRADE | Can be recommended according to individual circumstances | _ |
| Do not routinely use augmentation of an antidepressant with pindolol or thyroid hormones because there is no consistent evidence of efficacy. | [5] | GRADE | _ | _ |
| Thyroid hormones can be considered^*^  ^*^[7] recommends liothyronine. | [7] | MODIFIED GRADE | Highly recommended  Based on good-quality studies | Systematic reviews, meta-analyses, randomized trials, and health technology assessment reports |
|  | [6] | MODIFIED GRADE | Recommended with moderate clinical safety | _ |
| Bupropione, mianserin, modafinil, and triiodothyronine can be considered second-line adjuvant treatments. | [8] | OTHER | Recommended with moderate clinical safety | Level 2 |
| Methylphenidate and ziprasidone can be considered third-line adjuvant treatments. | [8] | OTHER | _ | Level 3 |
| Lisdexamphetamine and brexpiprazol can be considered second-line adjuvant treatments. | [8] | OTHER | _ | Level 1 |
| Tricyclic antidepressants, such as desipramine, can be considered third-line adjuvant treatments. | [8] | OTHER | _ | Level 2 |
