# Supplementary material for: Pharmacological treatment of depression: A systematic review comparing clinical practice guideline recommendations
Source: PLoS One. 2020 Apr 21;15(4):e0231700. doi: 10.1371/journal.pone.0231700 (PMC7173786; doi:10.1371/journal.pone.0231700)
Supplement: S3 Table — (DOCX) [file pone.0231700.s006.docx]

**S3 Table. Recommendations for the treatment of depression, extracted from clinical practice guidelines, listed for the elaboration of the synthesis published between January 2011 and April 2019: treatment for subtypes**

| **Recommendations** | **Clinical practice guidelines** | **Evidence classification scale** | **Recommendation strength** | **Level of evidence** |
| --- | --- | --- | --- | --- |
| **TREATMENT FOR SUBTYPES** | | | | |
| **CHRONIC DEPRESSION or DYSTHYMIA** | | | | |
| For patients with chronic depression, start with a combination of antidepressant drugs and psychotherapy | [9] | GRADE | Strong | High |
| Consider starting with drugs in patients with pure dysthymia | [9] | GRADE | Strong | High |
| For patients who do respond to initial treatment, consider augmenting drugs and adding psychotherapy | [9] | GRADE | Strong | High |
| **PSYCHOTIC DEPRESSION** | | | | |
| For psychotic patients, antipsychotics and antidepressants are recommended | [4] | GRADE | Strong | _ |
|  | [5] | GRADE | _ | _ |
|  | [6] | MODIFIED GRADE | Recommended with significant clinical safety | _ |
|  | [8] | OTHER | _ | Level 1 |
| In adult patients diagnosed with psychotic depression, it is not recommended to use antipsychotics as single therapy | [4] | GRADE | Strong | _ |
| Augmentation of lithium treatment may be helpful in patients who do not respond to the combination of antidepressants and antipsychotics | [6] | MODIFIED GRADE | _ | _ |
| It is recommended, preferably, to use tricyclic antidepressants for adult patients | [4] | GRADE | Weak | _ |
| **CATATONIC DEPRESSION** | | | | |
| Benzodiazepines are a treatment option, combined with antidepressant drugs | [8] | OTHER | _ | Level 3 |
|  | [6] | MODIFIED GRADE | Recommended with significant clinical safety | _ |
| Barbiturates are a treatment option, combined with antidepressant drugs | [6] | MODIFIED GRADE | Recommended with moderate clinical safety | _ |
| Catatonic patients may be more sensitive to neuroleptic malignant syndrome; therefore, careful monitoring of patients taking antipsychotics is required | [6] | MODIFIED GRADE | Recommended with moderate clinical safety | _ |
| **DEPRESSION WITH ATYPICAL CHARACTERISTICS** | | | | |
| No antidepressant has demonstrated superior efficacy | [8] | OTHER | _ | Level 2 |
| Monoamine oxidase inhibitors can be particularly effective. Consider a nutritional and/or psychiatric appointment when prescribing these drugs. | [9] | GRADE | _ | _ |
| Monoamine oxidase inhibitors are more effective than tricyclic antidepressants | [6] | MODIFIED GRADE | _ | _ |
| Selective serotonin reuptake inhibitors and bupropion can be used | [6] | MODIFIED GRADE | _ | _ |
| **DEPRESSION WITH MELANCHOLIC CHARACTERISTICS** | | | | |
| No antidepressant has demonstrated superior efficacy | [8] | OTHER | _ | Level 2 |
| Serotonin and noradrenaline reuptake inhibitors and tricyclic antidepressants may present some advantage over selective serotonin reuptake inhibitors | [6] | MODIFIED GRADE | _ | _ |
| **DEPRESSION WITH SEASONAL PATTERN** | | | | |
| No antidepressant has demonstrated superior efficacy | [8] | OTHER | _ | Level 2 and Level 3 |
| **DEPRESSION WITH SOMATIC SYMPTOMS (FATIGUE)** | | | | |
| Bupropion | [8] | OTHER | _ | Level 1 |
| Selective serotonin reuptake inhibitors | [8] | OTHER | _ | Level 2 |
| **MIXED DEPRESSION DISORDER** | | | | |
| Lurasidone and ziprasidone are treatment options | [8] | OTHER | _ | Level 2 and 3 |
